# A unifying mechanism for seipin‐mediated lipid droplet formation

**DOI:** 10.1002/1873-3468.14825

**Published:** 2024-02-13

**Authors:** Yoel A. Klug, Joana V. Ferreira, Pedro Carvalho

**Affiliations:** ^1^ Sir William Dunn School of Pathology University of Oxford UK

**Keywords:** endoplasmic reticulum, lipid droplet, neutral lipids, seipin, triglyceride

## Abstract

Lipid droplets (LDs) are dynamic organelles essential for cellular lipid homeostasis. Assembly of LDs occurs in the endoplasmic reticulum (ER), and the conserved ER membrane protein seipin emerged as a key player in this process. Here, we review recent advances provided by structural, biochemical, and *in silico* analysis that revealed mechanistic insights into the molecular role of the seipin complexes and led to an updated model for LD biogenesis. We further discuss how other ER components cooperate with seipin during LD biogenesis. Understanding the molecular mechanisms underlying seipin‐mediated LD assembly is important to uncover the fundamental aspects of lipid homeostasis and organelle biogenesis and to provide hints on the pathogenesis of lipid storage disorders.

## Graphical abstract

Seipin is a key factor in the assembly of lipid droplets at the endoplasmic reticulum. Here, we review recent advances provided by structural, biochemical, and *in silico* analyses that provided mechanistic insights into seipin function in lipid droplet formation.

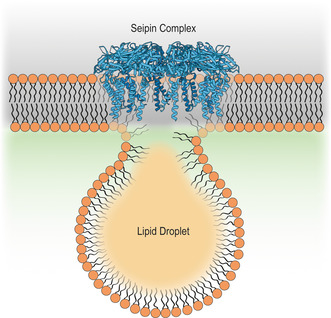

## Abbreviations

**CE**, cholesteryl esters

**EM**, electron microscopy

**ER**, endoplasmic reticulum

**FIT2**, fat‐induced transcript 2

**GPAT**, glycerol‐3‐phosphate acyltransferase

**LD**, lipid droplet

**LDAF1**, lipid droplet assembly factor 1

**LDAP**, lipid droplet‐associated proteins

**LDIP**, lipid droplet interacting proteins

**Ldo16/45**, lipid droplet organization proteins of 16 kDa and 45 kDa

**MD**, molecular dynamics

**NL**, neutral lipid

**NPC2**, niemann‐pick C2

**PA**, phosphatidic acid

**PPVs**, preperoxisomal vesicles

**SE**, steryl ester

**TAG**, triacylglycerol

**TM**, transmembrane

**VAP**, vesicle‐associated membrane protein‐associated protein

Lipid droplets (LDs) are evolutionary conserved organelles and the main cellular energy reservoir of eukaryotic cells by storing neutral lipids (NLs) in their core. These NLs, such as triacylglycerol (TAG) and steryl esters (SEs), can also be used to produce lipid precursors for the synthesis of new membranes. Hence, LDs emerge as crucial metabolic hubs that store energy and membrane building blocks. LDs can likewise mitigate lipotoxicity by diverting excess free fatty acids into NLs through esterification. Thus, the regulation of biogenesis, maintenance, and consumption of LDs is vital for lipid homeostasis and cellular metabolism [1, 2, 3, 4].

## How do lipid droplets form?

**Fig. 1:**
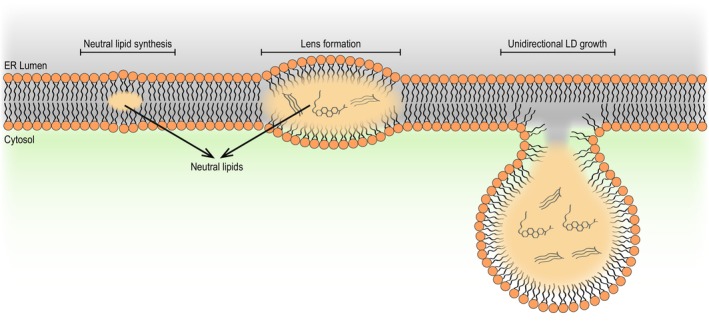
Lipid droplet biogenesis at the endoplasmic reticulum. Neutral lipids (NLs) are synthesized within the endoplasmic reticulum (ER) bilayer. Upon reaching a critical concentration, the NLs demix and coalesce to form a lens‐like structure. As NLs continue to be synthesized, the lens grows into a premature lipid droplet (LD). Upon continued growth, the LD buds toward the cytosolic face of the ER as a result of unidirectional growth. LDs can remain associated with the ER membrane or detach completely.

Among the cellular organelles, LDs have a unique structure composed of a NL core surrounded by a phospholipid monolayer [5, 6, 7]. LDs are assembled in the endoplasmic reticulum (ER), where both NLs and monolayer phospholipids are synthesized. At low concentrations, NLs are dissolved within the ER; however, if their concentration reaches 5–10%, they phase‐separate and form a lens‐like structure, the precursor of a new LD (Fig. 1) [8, 9, 10]. Further accumulation of NLs supports the growth of a nascent LD that eventually buds from the ER toward the cytosol to form a mature LD (Fig. 1) [1, 2, 3, 4, 7, 11]. In this model, the simple accumulation of NLs is necessary and sufficient to trigger LD formation. In fact, in both yeast and mammalian cells, LDs are completely lost only if NL synthesis is blocked, either genetically or pharmacologically [12, 13, 14]. Similarly, when NLs and aqueous solutions are mixed *in vitro*, particles with the structure and morphology of LDs are produced [15]. LD formation is also influenced by the membrane biophysical properties stemming from the phospholipid composition, since phospholipids that promote either negative or positive curvature have been shown to interfere with NL nucleation and LD budding [15, 16]. Also, a variety of proteins regulate the formation of LDs. Acting at different assembly steps, these proteins have an impact on the morphology, proteome, and lipidome of LDs ([17] and reviewed in [2, 3, 18]).

The main factor involved in LD biogenesis is seipin, an evolutionarily conserved ER integral membrane protein, which is mutated in patients with Berardinelli–Seip congenital lipodystrophy, a severe form of congenital generalized lipodystrophy [19]. In the budding yeast *Saccharomyces cerevisiae*, seipin (Sei1, also known as Fld1) and its functional partner Ldb16 were identified by genetic screens as mutants with aberrant LD morphology [20, 21]. In the absence of functional seipin, LDs still form but are highly heterogeneous, assembling in either small clusters or in a few supersized LDs [19, 20, 21]. These defects can arise from impaired LD maturation [22], defects in ER–LD contacts [23, 24], and an abnormal LD proteome [23]. Although almost two decades have passed since the contribution of seipin to LD formation was first described, the mechanism by which it facilitates LD assembly has started to unravel only recently [25, 26, 27, 28, 29, 30, 31]. Here, we summarize the latest advances in understanding the function of seipin and how it contributes to LD biogenesis.

### Seipin is central to LD homeostasis

**Fig. 2:**
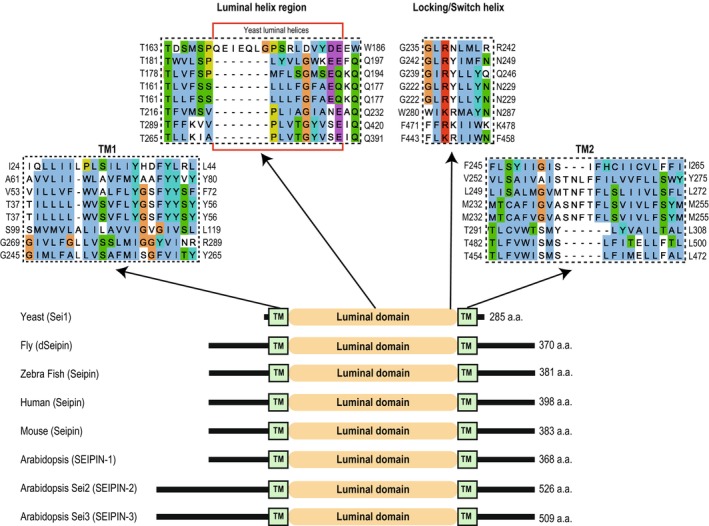
Seipin is an evolutionarily conserved protein. A schematic depiction of seipin morphology and multiple sequence alignments of specified domains in the designated species. Sequences were aligned using the Multiple Sequence Comparison by Log‐Expectation (MUSCLE) [75] and then depicted for graphical view by Jalview [76]. The red box in the luminal helix region represents the luminal helices in yeast Sei1. Yeast Sei1 was used as the reference sequence for the alignment, and the aligned sequences are in the same order depicted in the schematic below. Seipin sequences were taken from the following Uniprot entry numbers: Yeast – Q06058; Fly – Q9V3X4; Zebrafish – A0A8M2BKD6; Human – Q96G97 (isoform 1); Mouse – Q9Z2E9; Arabidopsis – SEI1 Q9FFD9, SEI2 F4I340, and SEI3 Q8L615.

Seipin is an evolutionarily conserved ER membrane protein composed of two transmembrane (TM) domains proximal to the N‐ and C‐termini and separated by an extended ER luminal domain (Fig. 2) [32, 33, 34]. In yeast, Ldb16, a fungi‐specific ER‐resident membrane protein, has been characterized as an obligatory binding partner of Sei1 and essential for a functional seipin complex [23, 35, 36]. Since then, seipin complexes with other protein partners have been characterized in most common model systems.

Seipin is localized to the ER membrane as foci that often correspond to subdomains for organelle biogenesis or ER–LD junctions [20, 23, 24, 36, 37, 38, 39, 40]. These have been suggested to be preferentially localized in ER tubules rather than ER sheets [41]. Immobilization of seipin in the nuclear envelope resulted in LD accumulation in this region [14], supporting the model that seipin determines the sites for LD biogenesis. Seipin has also been implicated in the biogenesis of nuclear LDs [42]. This topic will not be discussed here, as it was covered in detail in a recent review [43]. Several other ER proteins colocalize with seipin at the sites of LD formation, including the phosphatidic acid phosphatase Pah1 and its activators Nem1/Spo7; NLs biosynthetic enzymes, which promote localized synthesis of TAG [44, 45, 46]; the Fat Induced Transcript 2 (FIT2) proteins [47, 48, 49]; and Pex30 [38, 39]. Additionally, the Lipid Droplet Organization proteins of 16 and 45 kDa (Ldo16 and Ldo45, respectively) in yeast and the Ldo45 human homolog Promethin/lipid droplet assembly factor 1 (LDAF1) are recruited to the sites of LD biogenesis through their interaction with seipin [40, 50, 51, 52, 53]. How most of the proteins listed above contribute to LD formation requires further investigation. However, these proteins appear to play a regulatory role, while seipin has a central role in LD biogenesis.

Although seipin localizes in the ER membrane, it influences the properties of the LD surface. For example, seipin deletion promotes the recruitment of proteins to the LD monolayer that have amphipathic helices and lipid packing defect sensing motifs [23, 54, 55]. Seipin may also interfere with the flux of phospholipids from the ER into the LD monolayer. Therefore, by being localized at the ER–LD interface, seipin is well positioned to regulate the trafficking of proteins and lipids from the ER to the LDs [23, 24, 54]. Seipin has also been implicated in the metabolism of phosphatidic acid (PA), a precursor of TAG [35, 56, 57]. Yeast mutants lacking either seipin or Ldb16 accumulate increased levels of PA in ER regions adjacent to clusters of abnormal LDs. Interestingly, inhibition of phosphatidylcholine synthesis reverted this phenotype, leading to the formation of supersized LDs, highlighting the importance of ER phospholipid homeostasis in controlling LD size [57]. Seipin was shown to bind PA *in vitro* [25] and was also proposed to regulate glycerol‐3‐phosphate acyltransferase (GPAT) enzymes involved in the synthesis of PA [58]. The extent to which these observations contribute to regulating PA homeostasis at the sites of LD biogenesis should be addressed in the future.

In addition to its key role in LD biogenesis, seipin remains associated with the ER–LD interface, where it appears to control LD size. This role of seipin in LD maintenance was revealed in experiments in which the seipin protein was acutely depleted, resulting in a reduction in the number of small LDs and a rise in the number of supersized LDs. This observation has suggested that NLs diffused from smaller LDs with higher internal pressure to larger ones through the ER, as suggested by the Oswald ripening process [14]. Therefore, seipin can be envisioned to function as a valve that controls the flow of NLs, and eventually monolayer phospholipids and surface proteins, into and out of the LDs following their biogenesis.

## Structural insights into the seipin complexes

Recent cryo‐electron microscopy (cryo‐EM) structures of seipin from multiple species, together with molecular dynamic (MD) simulations and cell biology, provided tremendous insight into the mechanism by which seipin promotes LD formation.

### A lipid‐binding helix to concentrate TAG

**Fig. 3:**
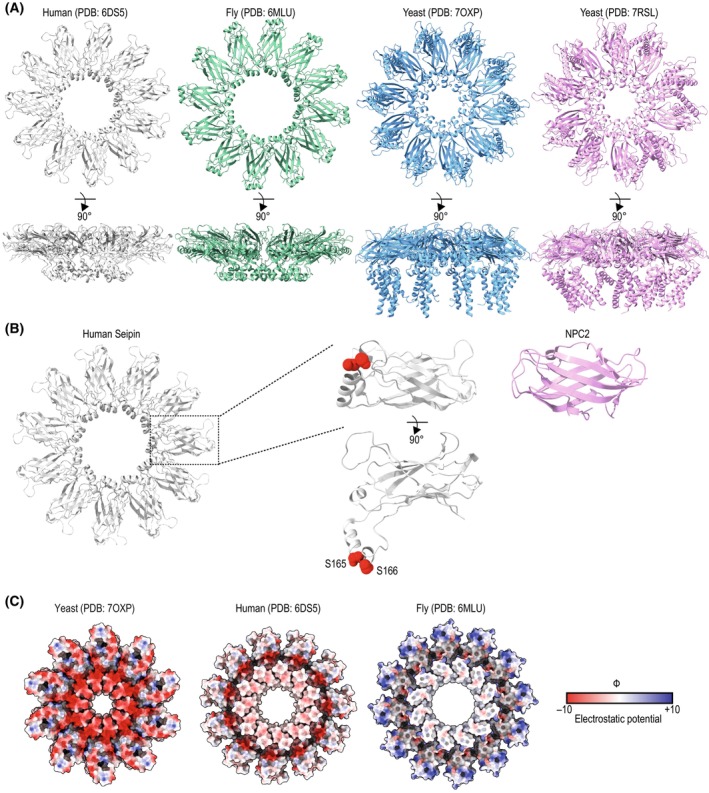
Seipin structure and function. (A) Cryogenic electron microscopy structures of seipin obtained from different model systems. Left to right: seipin from human (PDB: 6DS5 [25]), fly (PDB: 6MLU [26]), and yeast (PDB: 7OXP [27] and PDB: 7RSL [29]). The top exhibits a view from the cytosol toward the ER lumen, and the bottom corresponds to a 90° rotation. (B) Luminal domain of human seipin (PDB: 6DS5 [25]). The inset focuses on a protomer with the two serine residues S165 and S166 that are suggested to bind neutral lipids in red. Serine residues are positioned at the center of the seipin ring. On the right, the structure of the sterol‐binding protein Niemann‐Pick disease C2 (NPC2) (PDB: 2HKA [60]). NPC2 and the luminal domain of seipin share a β‐sheet fold, suggesting it can serve as a lipid‐binding motif. (C) Coulombic electrostatic potential of seipin luminal domains from yeast (PDB: 7OXP [27]), humans, and fly (as denoted in (A)). In contrast to human and fly seipin, the center of the yeast seipin ring is highly charged.

Structures of the luminal domains of human [25] and fly [26] seipin were determined by cryo‐EM (Fig. 3A). These studies revealed that seipin assembles into a defined homooligomeric ring consisting of 11 subunits in human cells [25] and 12 in flies [26], confirming earlier biochemical analysis in yeast that suggested seipin oligomers assembled as a toroid [59]. Mutations disrupting seipin oligomerization displayed strong defects in LD morphology, indicating that the assembly of the ring‐like structures is essential for seipin function [25, 26]. In the oligomer, each protomer of the luminal domain consists of a β‐sandwich fold. Curiously, this fold resembles lipid‐binding domains, such as the Niemann‐Pick C2 (NPC2) sterol‐binding domain [60], suggesting a potential lipid‐binding activity (Fig. 3B). In fact, the purified luminal domain of human seipin appears to bind to PA *in vitro* [25]. However, it is unclear whether this also occurs *in vivo*.

In addition to the β‐sandwich, the luminal domain of human and fly seipin shares a structural motif consisting of a hydrophobic helix lining the center of the seipin ring (Fig. 3A,C). A peptide composed of the fly seipin hydrophobic helix was shown to localize to LDs *in vivo* and *in vitro*. This localization was lost with a mutant peptide incorporating three aspartate residues, suggesting that this helix may take advantage of the packing defects present on the LD surface and binding NLs [26]. Further support for the role of this hydrophobic helix in TAG binding came from MD simulations [61]. When embedded in a lipid bilayer with a composition that mimics the ER, the luminal domain of human seipin was able to interact with TAG even if it was present at very low concentrations [30, 61]. Given that the hydrophobic helix sits in the center of the seipin oligomeric ring, the binding leads to an effective concentration of TAG molecules at its center, thereby facilitating their phase separation. The simulations indicate that two conserved serine residues, via their hydroxyl group, mediate the interactions with carboxyl ester groups of TAG [28, 30] (Fig. 3B). Consistent with *in silico* experiments, mutations of these serine residues showed defects in the LD morphology. More recently, similar experiments revealed that seipin uses a similar chemistry to concentrate other NLs, such as cholesteryl esters (CE), since the hydroxyl groups in seipin interact with the carboxyl ester groups present in NLs [61]. Consistent with these observations in yeast, seipin‐deficient mice show reduced CE‐containing LDs in steroidogenic tissues [62]. These findings contrast with earlier studies suggesting that seipin was dispensable for the formation of LDs containing CE and retinyl esters, another type of neutral lipid [63]. The causes for the discrepancy are unclear, but, given that CE has a much higher melting temperature when compared to TAG (44C vs. 4C, respectively), they may be attributed at least in part to the different temperatures at which the experiments were conducted in the two studies. It has been shown that, given their high melting temperature, the packaging of CE into LDs can also be facilitated by TAG, which can act as a solvent even if present in trace amounts [64]. Thus, it is possible that minute levels of TAG can also facilitate the nucleation of CE under certain conditions.

**Fig. 4:**
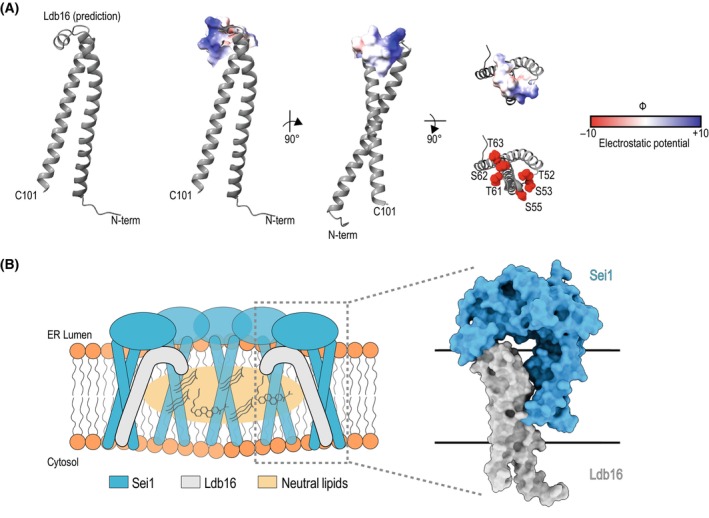
Ldb16 complements Sei1 for TAG binding. (A) TrRosetta [77] based model of Ldb16 (residues 1–101) is predicted to encompass both transmembrane domains and the luminal helix. The Coulombic electrostatic potential of the luminal helix is depicted. Ldb16 presents an electrostatic neutral face, possibly toward the center of the yeast seipin ring. This may potentially create a similar electrostatic neutral interface similar to human and fly seipin that may serve as the neutral lipid‐binding site of the yeast seipin complex. Hydroxyl residues suggested to be important for neutral lipid binding are shown in red. (B) Hypothetical model of the Ldb16 position within the Sei1 ring. (Left) Speculative schematic of how yeast Ldb16 resides within the seipin disc to promote neutral lipid accumulation. (Right) Structural depiction of how a protomer of Sei1 and of Ldb16 might sit together. Sei1 is based on PDB: 7OXP [27] and Ldb16 is a predicted model as explained in (A). The membrane bilayer is denoted by black lines.

More recently, two structures of the full‐length yeast seipin were also solved (Fig. 3A) [27, 29]. Like human and fly seipin, yeast seipin forms a homooligomeric ring comprised of a β‐sandwich fold, but with only 10 subunits. However, instead of a hydrophobic luminal helix, the yeast seipin luminal helix is polar and shorter (Fig. 3C). As expected for a polar helix, MD simulations revealed that this feature is ineffective in binding TAG [27]. Yeast seipin overcomes this limitation through its binding partner, Ldb16 [23, 27, 36]. In fact, Ldb16 is an obligatory seipin partner and is unstable in the absence of Sei1 [27, 36]. Through a site‐specific photocrosslinking approach, the position of Ldb16 was mapped and shown to reside in the center of the yeast seipin ring [27]. Structural prediction and mutagenesis studies revealed that Ldb16 provides the missing hydrophobic helix harboring hydroxylated residues suitable for binding to TAG (Fig. 4A,B) [27, 61], similarly to human seipin. This suggests that in yeast, seipin function is broken down into two polypeptides. Consequently, human seipin can rescue a *sei1Δldb16Δ* phenotype [27, 36]. Taken together, these data support the model in which all seipin complexes use a similar molecular mechanism to concentrate NLs. Thus, a unifying molecular mechanism for seipin function emerges.

### Rearrangements within seipin complexes

**Fig. 5:**
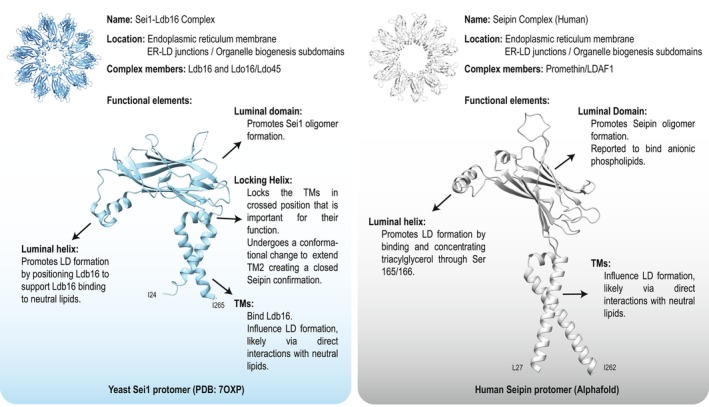
Reported seipin complex functional elements. Location, partners, and functional elements of yeast (left) and human isoform 1 (right) seipin. The functional elements are annotated on a protomer of Sei1 (PDB: 7OXP [27]) and human seipin (as predicted by Alphafold). Sei1‐TMs are modeled based on the electron density observed in PDB: 7OXP [27], hence shorter than predicted. Human seipin encompasses the full length of the TMs, as predicted by Alphafold.

A major advance of yeast seipin structures was that they allowed the first visualization of seipin transmembrane segments (Figs 3A and 5) [27, 29]. The yeast seipin TMs sit proximal to the short N‐ and C‐termini and are separated by an extended β‐sandwich. They also adopted a unique crossed arrangement that is stabilized by the TM2 capping helix, termed the locking helix (also called switch region) (Fig. 4) [27, 29]. Consequently, both *in vivo* experiments and MD simulations revealed that TM positioning plays a role in LD formation and TAG accumulation [27, 29, 31]. Structural, MD, and *in vivo* analysis showed that under the deletion of the locking helix, the TMs move in relation to one another, resulting in disturbed LD formation [27]. As noted beforehand and shown in Fig. 3, two similar structures or yeast seipin were reported [27, 29]. While in one of the structures, the 10 protomers of the seipin ring appeared identical (PDB: 7OXP), the second structure showed protomers alternating between two distinct conformations, called A and B (PDB: 7RSL). The A conformation was similar to the one described above. In the B conformation, the locking helix rotates to become part of an extended TM2, with both TMs displaying an increased tilt and projecting toward the center of the ring in a cage‐like organization [27, 29]. It was proposed that the cage‐like arrangement would further facilitate TAG concentration to support LD budding and growth. Further investigation will be required to validate the presence of multiple seipin conformations within the same ring, as this was not observed in other structural or *in silico* studies. Moreover, it is unclear how the alternating conformations would function in a seipin ring with an odd number of protomers, such as in humans (11 protomers per seipin ring).

The exact mechanism of how the TMs affect LD formation remains unclear. Previous MD simulations conducted on human seipin pinpointed specific regions of the TMs that may play a specific role in TAG accumulation [30]. However, this was performed with a naive placement of the TMs within the bilayer before the TMs of yeast seipin were resolved and *in silico* structure prediction was available. With higher resolution seipin structures available, as well as *in silico* structure prediction, it may be possible to understand how the TMs of seipin promote efficient LD formation. This approach was recently used to examine seipin‐mediated LD formation in a large MD system where TAG spontaneously nucleates [31]. Interestingly, simulations of LD budding with the human seipin with or without its TMs showed that the TMs pushed TAG toward the budding LD and facilitated the emergence of the membrane stalk connecting the ER and LDs [14]. Surprisingly, as the simulations were extended in time, the TMs shifted and adopted an open conformation [31]. This TM opening was also observed in a recent MD study looking at the initial stages of LD formation. However, in this study, seipin was not sufficient to induce directional budding in tubular membranes. Moreover, directional budding was lost when the TM opening occurred [65]. It has been postulated that this tilting of the TMs could support the growth of LDs and stabilize the contact with a mature LD [66]. While these data support a role for seipin TMs during LD formation, a comprehensive understanding of seipin function and dynamics during the several stages of LD biogenesis is still lacking.

## Seipin‐interacting proteins

Studies searching for binding partners of yeast seipin led to the identification of Lipid Droplet Organization proteins of 16 and 45 kDa (Ldo16 and Ldo45, respectively) [51, 52, 58]. Notably, these two proteins are encoded by a consecutive, partly overlapping open reading frame and are generated by alternative splicing with the Ldo16 sequence included in the Ldo45 sequence. Both Ldo16 and Ldo45 bind to the seipin complex independently of each other, and their deletion results in a mild LD morphology defect [51, 52]. Therefore, it has been hypothesized that they might act as regulatory subunits of the seipin complex.

Ldo16 and Ldo45 appear to have common and distinct functions. Ldo45 favors LD growth and TAG accumulation, while Ldo16 appears to function primarily during LD consumption through lipophagy [51, 52]. In fact, recently, a new role of mediating LD tethering to the vacuole was described for Ldo16 due to its interaction with the vacuolar protein Vac8 [67, 68]. In the absence of Ldo16, Ldo45 can also interact with Vac8 [67, 68]. As Ldo45 encompasses Ldo16, it is plausible that the ability of Ldo16 to target LDs in the vacuole is retained in Ldo45. These studies are beginning to shed light on the molecular role of Ldo16 in the LD life cycle. However, the molecular role of Ldo45 remains a mystery.

Based on sequence similarity and immunoprecipitation experiments, the human protein LDAF1 (also known as Promethin/TMEM159 or CG32803 in fly) was proposed to be homologous to the yeast Ldo45 [50, 53]. Immunoprecipitation experiments in mammalian cultured cells showed that LDAF1 binds to seipin. Curiously, this interaction requires a seipin hydrophobic helix, which is also important to bind and concentrate TAG within the seipin ring [28, 40]. In yeast, Sei1, Ldb16, and Ldo45 were shown to form a complex, although the regions involved in complex assembly and recruitment of Ldo45 have not been defined. Furthermore, LDAF1 was suggested to regulate the morphology of LDs and the accumulation of TAG [40], similarly to Ldo45 [51, 52], but the exact molecular role of Ldo45 and LDAF1 remains unclear. Following the advances in the molecular mechanisms of the seipin complexes succeeding the cryo‐EM structures, future work on the structure of the complete seipin complexes with their interactors may help us to understand how these proteins contribute to LD homeostasis.

Other proteins seem to interact or collaborate with seipin complexes in the regulation of lipid metabolism. An example is the yeast ER‐resident protein, Pex30, and its human homolog, MCTP2. These have been characterized as factors that contribute to LD biogenesis and LD maintenance [38, 39, 58, 69, 70]. The role of Pex30 is especially important in the absence of the seipin complex, since *sei1Δpex30Δ* cells are unable to produce new LDs or preperoxisomal vesicles (PPVs), the precursors of new peroxisomes. Therefore, a high lipotoxic effect is observed as a strong growth defect [39]. Together with Sei1, Pex30 contributes to the formation of ER subdomains for the generation of new LDs and PPVs [38, 39, 45].

In developing adipocytes, seipin has been reported to interact with AGPAT2, Lipin‐1 [71], and GPAT3 [58], which are enzymes belonging to the lipid synthesis pathway. This interaction is suggested to facilitate adipocyte differentiation [71, 72].

*Arabidopsis thaliana* contains three seipin isoforms (SEIPIN‐1, ‐2, ‐3) that were reported to collaborate for the normal number and size of LDs with two LD proteins: LDAPs (LD‐associated proteins) and LDIP (LDAP‐interacting protein). Interestingly, LDIP, which was proposed to have an analogous function to LDAF1/Promethin, interacts with seipin through its conserved hydrophobic helix [73]. However, only SEIPIN‐2 and SEIPIN‐3 interact with VAP27‐1 (vesicle‐associated membrane protein‐Associated Protein 27‐1), a membrane contact site protein with a lipid transfer role [74].

## Conclusions and future directions

Over the past few decades, the advancement in understanding of LDs, from the idea of simple lipid accumulation to a well‐regulated organelle critical to cellular homeostasis, has come a long way. In recent years, the mechanism of LD formation has taken shape, with seipin taking center stage. Recent structural data on seipin complexes have provided much clarification on the contribution of the distinct protein domains of seipin in LD formation (Fig. 5). In fact, it is becoming evident that the molecular mechanisms by which seipin complexes promote LD formation are extremely conserved.

In the future, it will be important to understand the differences in seipin structure and interactors among different species, as well as how seipin partners and other associated factors coordinate their activities during the various stages of the life cycle of LDs: from monolayer organization to LD growth and shrinkage, including participation in protein diffusion and targeting. As in recent years, these insights will likely come from the intersection of imaging, structural, and modeling approaches and may contribute to the development of treatments against LD‐related pathologies.
